# Tetra­methyl­ammonium dihydrogen phosphate hemihydrate

**DOI:** 10.1107/S1600536809009179

**Published:** 2009-03-19

**Authors:** Kyoko Fujita, Douglas R. MacFarlane, Keiichi Noguchi, Hiroyuki Ohno

**Affiliations:** a Department of Biotechnology, Tokyo University of Agriculture and Technology, 2-24-16 Naka-cho, Koganei, Tokyo 184-8588, Japan; b School of Chemistry, Monash University, Wellington Road, Clayton, Victoria 3800, Australia; c Instrumentation Analysis Center, Tokyo University of Agriculture and Technology, 2-24-16 Naka-cho, Koganei, Tokyo 184-8588, Japan

## Abstract

In the crystal structure of the title compound, C_4_H_12_N^+^·H_2_PO_4_^−^·0.5H_2_O, the anions form an infinite hydrogen-bonded chain along the [1
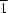
0] direction. The anion chains are connected by water mol­ecules, which lie on crystallographic twofold rotation axes, through O—H⋯O hydrogen bonds. These hydrogen bonds are almost perpendicular to the other hydrogen bonds which create an assembled structure of anions. In addition, C—H⋯O hydrogen bonds between anions and cations are observed.

## Related literature

For the structure of tetra­methyl­ammonium dihydrogen phosphate monohydrate, see: Ohama *et al.* (1987 ▶).
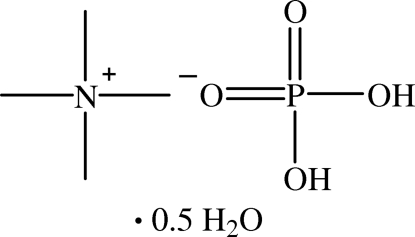


## Experimental

### 

#### Crystal data

C_4_H_12_N^+^·H_2_PO_4_^−^·0.5H_2_O
*M* *_r_* = 180.14
Monoclinic, 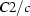

*a* = 14.3213 (3) Å
*b* = 9.2607 (2) Å
*c* = 13.1990 (2) Å
β = 103.614 (1)°
*V* = 1701.34 (6) Å^3^
*Z* = 8
Cu *K*α radiation
μ = 2.72 mm^−1^
*T* = 193 K
0.40 × 0.35 × 0.20 mm

#### Data collection

Rigaku R-AXIS RAPID diffractometer
Absorption correction: numerical (**NUMABS**; Higashi, 1999 ▶) *T*_min_ = 0.390, *T*_max_ = 0.580
14805 measured reflections
1565 independent reflections
1505 reflections with *I* > 2σ(*I*)
*R*_int_ = 0.040

#### Refinement

*R*[*F* ^2^ > 2σ(*F* ^2^)] = 0.032
*wR*(*F* ^2^) = 0.084
*S* = 1.05
1565 reflections
113 parameters
H atoms treated by a mixture of independent and constrained refinement
Δρ_max_ = 0.23 e Å^−3^
Δρ_min_ = −0.31 e Å^−3^

### 

Data collection: *PROCESS-AUTO* (Rigaku/MSC, 1998 ▶); cell refinement: *PROCESS-AUTO*; data reduction: *CrystalStructure* (Rigaku/MSC, 2004 ▶); program(s) used to solve structure: *SIR2004* (Burla *et al.*, 2005 ▶); program(s) used to refine structure: *SHELXL97* (Sheldrick, 2008 ▶); molecular graphics: *ORTEPIII* (Burnett & Johnson, 1996 ▶); software used to prepare material for publication: *SHELXL97*.

## Supplementary Material

Crystal structure: contains datablocks global, I. DOI: 10.1107/S1600536809009179/is2395sup1.cif

Structure factors: contains datablocks I. DOI: 10.1107/S1600536809009179/is2395Isup2.hkl

Additional supplementary materials:  crystallographic information; 3D view; checkCIF report

## Figures and Tables

**Table 1:** Hydrogen-bond geometry (Å, °)

*D*—H⋯*A*	*D*—H	H⋯*A*	*D*⋯*A*	*D*—H⋯*A*
O3—H3O⋯O2^i^	0.96 (4)	1.57 (4)	2.5196 (18)	169 (4)
O4—H4O⋯O1^ii^	0.75 (3)	1.83 (3)	2.5644 (19)	169 (3)
O5—H5O⋯O1	0.83 (3)	2.06 (3)	2.8883 (15)	173 (3)
C1—H1*B*⋯O1^iii^	0.98	2.62	3.405 (2)	137
C2—H2*B*⋯O4^iv^	0.98	2.39	3.291 (3)	153
C2—H2*C*⋯O2	0.98	2.59	3.506 (3)	156
C2—H2*C*⋯O1	0.98	2.62	3.473 (3)	145
C3—H3*A*⋯O3^v^	0.98	2.57	3.495 (3)	157
C4—H4*C*⋯O3^vi^	0.98	2.62	3.465 (3)	144

Symmetry codes: (i) 
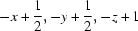
; (ii) 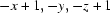; (iii) 
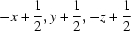
; (iv) 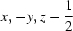; (v) 
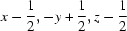
; (vi) 
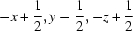
.

**Fig. 1.**
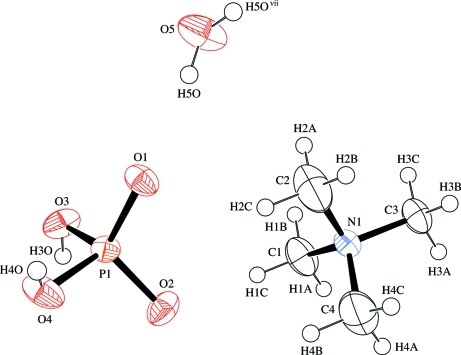
Displacement ellipsoid plot and atomic numbering scheme of (I). Ellipsoids are drawn at the 50% probability level and H atoms are shown as small spheres of arbitrary radii. [Symmetry code: (vii) -x + 1, y, -z + 1/2.]

**Fig. 2.**
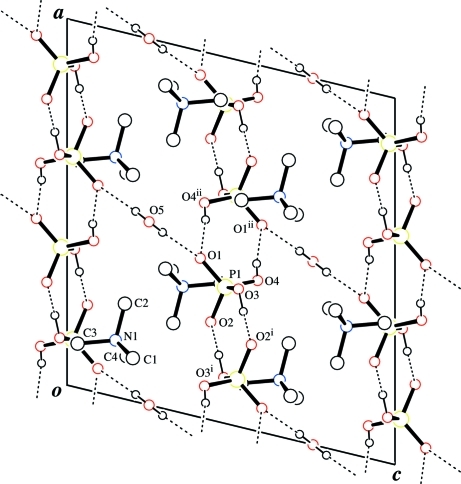
The molecular packing of (I), viewed along the b axis. Dashed lines indicate intermolecular O—H···O hydrogen bonds. For clarity, only H atoms involved in O—H···O hydrogen bonding have been included. [Symmetry codes: (i) -x + 1/2,-y + 1/2, -z + 1; (ii) -x + 1, -y, -z + 1.]

**Fig. 3.**
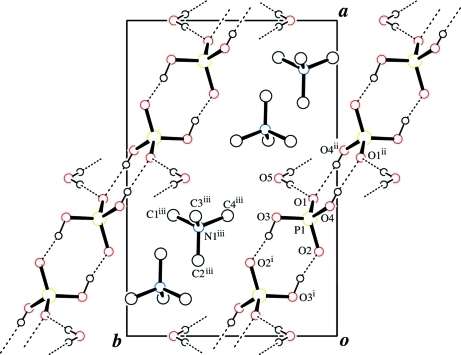
The molecular packing of (I), viewed along the c axis. Dashed lines indicate intermolecular O—H···O hydrogen bonds. For clarity, only H atoms involved in O—H···O hydrogen bonding have been included. [Symmetry codes: (i) -x + 1/2,-y + 1/2, -z + 1; (ii) -x + 1, -y, -z + 1; (iii) -x + 1/2, y + 1/2, -z + 1/2.]

## supplementary crystallographic information

### Comment

The title compound, (I), forms two hydrate states, hemihydrate and monohydrate. Basic structure about monohydrate had been published (Ohama *et al.*, 1987). We report herein the crystal structure of the hemihydrate compound.

The molecular structures of (I) are shown in Fig. 1. There are eight anions and cations, and four water molecules in a unit cell. The water molecules are located on twofold rotation axes. The anions create infinite chains by using two hydrogen bonds of O4—H···O1 and O3—H···O2 (Fig. 2). These chains run two different directions mutually along the *c* axis. One is [110] direction, the other is [110] direction. Water molecules connect the anion chains by hydrogen bonds of O5—H···O1, so as to create three dimensional networks. The cations are arranged along with the anion chains (Fig. 3). Molecular packing is additionally stabilized by C—H···O hydrogen bonds between anions and cations (Table 1).

### Experimental

Tetramethylammonium hydrated solution was mixed with phosphoric acid. The solvent was evaporated and product was dried in vacuo. Final purification was achieved by recrystallization from a methanol solution. The compound was identified using ^1^H NMR, DSC and Electrospray mass spectrometry.

### Refinement

Hydroxyl H atoms in dihydrogen phosphate and water molecule were located in a difference Fourier map and were subsequently refined freely. Methyl H atoms were positioned by using the HFIX 137 instruction in *SHELXL97*, with C—H = 0.98 Å and *U*_iso_(H) = 1.2*U*_eq_(C).

### Crystal data

**Table:**

C_4_H_12_N^+^·H_2_O_4_P^−^·0.5H_2_O	*F*(000) = 776
*M**_r_* = 180.14	*D*_x_ = 1.407 Mg m^−^^3^
Monoclinic, *C*2/*c*	Cu *K*α radiation, λ = 1.54187 Å
Hall symbol: -C 2yc	Cell parameters from 13463 reflections
*a* = 14.3213 (3) Å	θ = 3.4–68.2°
*b* = 9.2607 (2) Å	µ = 2.72 mm^−^^1^
*c* = 13.1990 (2) Å	*T* = 193 K
β = 103.614 (1)°	Block, colorless
*V* = 1701.34 (6) Å^3^	0.40 × 0.35 × 0.20 mm
*Z* = 8	

### Data collection

**Table:**

Rigaku R-AXIS RAPID diffractometer	1565 independent reflections
Radiation source: rotating anode	1505 reflections with *I* > 2σ(*I*)
graphite	*R*_int_ = 0.040
Detector resolution: 10.00 pixels mm^-1^	θ_max_ = 68.2°, θ_min_ = 5.7°
ω scans	*h* = −17→17
Absorption correction: numerical (*NUMABS*; Higashi, 1999)	*k* = −11→11
*T*_min_ = 0.390, *T*_max_ = 0.580	*l* = −15→15
14805 measured reflections	

### Refinement

**Table:**

Refinement on *F*^2^	Secondary atom site location: difference Fourier map
Least-squares matrix: full	Hydrogen site location: difference Fourier map
*R*[*F*^2^ > 2σ(*F*^2^)] = 0.032	H atoms treated by a mixture of independent and constrained refinement
*wR*(*F*^2^) = 0.084	*w* = 1/[σ^2^(*F*_o_^2^) + (0.0404*P*)^2^ + 2.3234*P*] where *P* = (*F*_o_^2^ + 2*F*_c_^2^)/3
*S* = 1.05	(Δ/σ)_max_ < 0.001
1565 reflections	Δρ_max_ = 0.23 e Å^−^^3^
113 parameters	Δρ_min_ = −0.31 e Å^−^^3^
0 restraints	Extinction correction: *SHELXL97* (Sheldrick, 2008), Fc^*^=kFc[1+0.001xFc^2^λ^3^/sin(2θ)]^-1/4^
Primary atom site location: structure-invariant direct methods	Extinction coefficient: 0.0039 (3)

### Special details

**Table:**

Geometry. All e.s.d.'s (except the e.s.d. in the dihedral angle between two l.s. planes) are estimated using the full covariance matrix. The cell e.s.d.'s are taken into account individually in the estimation of e.s.d.'s in distances, angles and torsion angles; correlations between e.s.d.'s in cell parameters are only used when they are defined by crystal symmetry. An approximate (isotropic) treatment of cell e.s.d.'s is used for estimating e.s.d.'s involving l.s. planes.
Refinement. Refinement of *F*^2^ against ALL reflections. The weighted *R*-factor *wR* and goodness of fit *S* are based on *F*^2^, conventional *R*-factors *R* are based on *F*, with *F* set to zero for negative *F*^2^. The threshold expression of *F*^2^ > σ(*F*^2^) is used only for calculating *R*-factors(gt) *etc*. and is not relevant to the choice of reflections for refinement. *R*-factors based on *F*^2^ are statistically about twice as large as those based on *F*, and *R*- factors based on ALL data will be even larger.

### Fractional atomic coordinates and isotropic or equivalent isotropic displacement parameters (Å^2^)

**Table:**

	*x*	*y*	*z*	*U*_iso_*/*U*_eq_	
P1	0.37195 (3)	0.13092 (4)	0.48223 (3)	0.02350 (19)	
O1	0.43439 (8)	0.11276 (14)	0.40616 (9)	0.0306 (3)	
O2	0.26771 (8)	0.08626 (14)	0.43819 (10)	0.0355 (3)	
O3	0.37576 (9)	0.28806 (13)	0.52247 (11)	0.0355 (3)	
O4	0.41159 (10)	0.03446 (16)	0.58089 (10)	0.0375 (4)	
N1	0.15455 (10)	0.15807 (15)	0.14754 (10)	0.0264 (3)	
C1	0.11725 (17)	0.2789 (2)	0.20042 (16)	0.0456 (5)	
H1A	0.0469	0.2784	0.1808	0.055*	
H1B	0.1410	0.3707	0.1793	0.055*	
H1C	0.1393	0.2675	0.2761	0.055*	
C2	0.26055 (16)	0.1614 (4)	0.17612 (19)	0.0726 (9)	
H2A	0.2831	0.2550	0.1566	0.087*	
H2B	0.2856	0.0841	0.1392	0.087*	
H2C	0.2833	0.1472	0.2515	0.087*	
C3	0.12037 (14)	0.1727 (2)	0.03190 (14)	0.0384 (5)	
H3A	0.0502	0.1653	0.0122	0.046*	
H3B	0.1484	0.0956	−0.0023	0.046*	
H3C	0.1401	0.2667	0.0100	0.046*	
C4	0.1185 (3)	0.0209 (3)	0.1815 (2)	0.0816 (10)	
H4A	0.0481	0.0194	0.1602	0.098*	
H4B	0.1390	0.0128	0.2576	0.098*	
H4C	0.1444	−0.0605	0.1493	0.098*	
O5	0.5000	0.2765 (2)	0.2500	0.0533 (6)	
H3O	0.318 (3)	0.325 (5)	0.537 (3)	0.130 (14)*	
H4O	0.4569 (19)	−0.003 (3)	0.5780 (19)	0.048 (7)*	
H5O	0.4803 (19)	0.223 (3)	0.291 (2)	0.059 (8)*	

### Atomic displacement parameters (Å^2^)

**Table:**

	*U*^11^	*U*^22^	*U*^33^	*U*^12^	*U*^13^	*U*^23^
P1	0.0201 (3)	0.0248 (3)	0.0272 (3)	0.00388 (15)	0.00872 (17)	−0.00004 (15)
O1	0.0281 (6)	0.0381 (7)	0.0282 (6)	0.0099 (5)	0.0117 (5)	0.0056 (5)
O2	0.0240 (6)	0.0379 (7)	0.0445 (7)	−0.0021 (5)	0.0074 (5)	−0.0135 (6)
O3	0.0291 (6)	0.0264 (7)	0.0538 (8)	−0.0022 (5)	0.0155 (6)	−0.0083 (6)
O4	0.0340 (7)	0.0466 (8)	0.0380 (7)	0.0179 (6)	0.0206 (6)	0.0146 (6)
N1	0.0293 (7)	0.0262 (7)	0.0246 (7)	0.0037 (6)	0.0081 (6)	0.0018 (5)
C1	0.0584 (13)	0.0441 (12)	0.0370 (10)	0.0183 (10)	0.0169 (9)	−0.0020 (9)
C2	0.0317 (11)	0.145 (3)	0.0396 (12)	0.0238 (14)	0.0061 (9)	−0.0052 (15)
C3	0.0414 (10)	0.0471 (11)	0.0260 (9)	0.0102 (9)	0.0064 (8)	0.0030 (8)
C4	0.162 (3)	0.0378 (13)	0.0494 (14)	−0.0344 (17)	0.0325 (17)	−0.0002 (11)
O5	0.0807 (17)	0.0291 (11)	0.0653 (15)	0.000	0.0476 (13)	0.000

### Geometric parameters (Å, °)

**Table:**

P1—O1	1.5029 (12)	C1—H1C	0.9800
P1—O2	1.5261 (12)	C2—H2A	0.9800
P1—O3	1.5456 (12)	C2—H2B	0.9800
P1—O4	1.5710 (13)	C2—H2C	0.9800
O3—H3O	0.96 (5)	C3—H3A	0.9800
O4—H4O	0.75 (3)	C3—H3B	0.9800
N1—C2	1.476 (3)	C3—H3C	0.9800
N1—C4	1.480 (3)	C4—H4A	0.9800
N1—C1	1.483 (2)	C4—H4B	0.9800
N1—C3	1.495 (2)	C4—H4C	0.9800
C1—H1A	0.9800	O5—H5O	0.83 (3)
C1—H1B	0.9800		
			
O1—P1—O2	113.43 (7)	H1B—C1—H1C	109.5
O1—P1—O3	110.89 (7)	N1—C2—H2A	109.5
O2—P1—O3	109.77 (7)	N1—C2—H2B	109.5
O1—P1—O4	109.53 (7)	H2A—C2—H2B	109.5
O2—P1—O4	106.97 (8)	N1—C2—H2C	109.5
O3—P1—O4	105.89 (8)	H2A—C2—H2C	109.5
P1—O3—H3O	116 (3)	H2B—C2—H2C	109.5
P1—O4—H4O	111.8 (19)	N1—C3—H3A	109.5
C2—N1—C4	110.6 (2)	N1—C3—H3B	109.5
C2—N1—C1	109.08 (18)	H3A—C3—H3B	109.5
C4—N1—C1	108.36 (18)	N1—C3—H3C	109.5
C2—N1—C3	109.20 (15)	H3A—C3—H3C	109.5
C4—N1—C3	109.46 (17)	H3B—C3—H3C	109.5
C1—N1—C3	110.14 (14)	N1—C4—H4A	109.5
N1—C1—H1A	109.5	N1—C4—H4B	109.5
N1—C1—H1B	109.5	H4A—C4—H4B	109.5
H1A—C1—H1B	109.5	N1—C4—H4C	109.5
N1—C1—H1C	109.5	H4A—C4—H4C	109.5
H1A—C1—H1C	109.5	H4B—C4—H4C	109.5

### Hydrogen-bond geometry (Å, °)

**Table:**

*D*—H···*A*	*D*—H	H···*A*	*D*···*A*	*D*—H···*A*
O3—H3O···O2^i^	0.96 (4)	1.57 (4)	2.5196 (18)	169 (4)
O4—H4O···O1^ii^	0.75 (3)	1.83 (3)	2.5644 (19)	169 (3)
O5—H5O···O1	0.83 (3)	2.06 (3)	2.8883 (15)	173 (3)
C1—H1B···O1^iii^	0.98	2.62	3.405 (2)	137
C2—H2B···O4^iv^	0.98	2.39	3.291 (3)	153
C2—H2C···O2	0.98	2.59	3.506 (3)	156
C2—H2C···O1	0.98	2.62	3.473 (3)	145
C3—H3A···O3^v^	0.98	2.57	3.495 (3)	157
C4—H4C···O3^vi^	0.98	2.62	3.465 (3)	144

Symmetry codes: (i) −*x*+1/2, −*y*+1/2, −*z*+1; (ii) −*x*+1, −*y*, −*z*+1;  (iii) −*x*+1/2, *y*+1/2, −*z*+1/2; (iv) *x*, −*y*, *z*−1/2; (v) *x*−1/2, −*y*+1/2, *z*−1/2; (vi) −*x*+1/2, *y*−1/2, −*z*+1/2.
